# Antinociceptive and Antipruritic Effects of HSK21542, a Peripherally-Restricted Kappa Opioid Receptor Agonist, in Animal Models of Pain and Itch

**DOI:** 10.3389/fphar.2021.773204

**Published:** 2021-11-16

**Authors:** Xin Wang, Xiaoli Gou, Xiaojuan Yu, Dongdong Bai, Bowei Tan, Pingfeng Cao, Meilin Qian, Xiaoxiao Zheng, Hairong Wang, Pingming Tang, Chen Zhang, Fei Ye, Jia Ni

**Affiliations:** ^1^ Intensive Care Unit, First Affiliated Hospital of Xinjiang Medical University, Urumqi, China; ^2^ Center for Drug Research and Development, Haisco Pharmaceutical Group Co., Ltd., Chegdu, China

**Keywords:** HSK21542, kappa opioid receptor, pain, pruritus, animal models

## Abstract

Kappa opioid receptor (KOR) agonists have been promising therapeutic candidates, owing to their potential for relieving pain and treating intractable pruritus. Although lacking morphine-like central nervous system (CNS) effects, KOR agonists do elicit sedation, dysphoria and diuresis which seriously impede their development. Peripherally-restricted KOR agonists have a poor ability to penetrate into the CNS system, so that CNS-related adverse effects can be ameliorated or even abolished. However, the only approved peripherally-restricted KOR agonist CR845 remains some frequent CNS adverse events. In the present study, we aim to address pharmacological profiles of HSK21542, with an expectation to provide a safe and effective alternative for patients who are suffering from pain and pruritus. The *in vitro* experimental results showed that HSK21542 was a selective and potent KOR agonist with higher potency than CR845, and had a brain/plasma concentration ratio of 0.001, indicating its peripheral selectivity. In animal models of pain, HSK21542 significantly inhibited acetic acid-, hindpaw incision- or chronic constriction injury-induced pain-related behaviors, and the efficacy was comparable to CR845 at 15 min post-dosing. HSK21542 had a long-lasting analgesic potency with a median effective dose of 1.48 mg/kg at 24 h post-drug in writhing test. Meanwhile, the antinociceptive activity of HSK21542 was effectively reversed by a KOR antagonist nor-binaltorphimine. In addition, HSK21542 had powerful antipruritic activities in compound 48/80-induced itch model. On the other hand, HSK21542 had a weak ability to produce central antinociceptive effects in a hot-plate test and fewer effects on the locomotor activity of mice. HSK21542 didn’t affect the respiratory rate of mice. Therefore, HSK21542 might be a safe and effective KOR agonist and promising candidate for treating pain and pruritus.

## Introduction

Kappa opioid receptor (KOR), one of the classical opioid receptors, is an inhibitory G-protein coupled receptor (GPCR) that is distributed in both the central nervous system (CNS) and the peripheral tissues (Ragen et al., 2015; Snyder et al., 2018). Due to the wide distribution and associated physiological functions of KOR, it has been used in exploring as a potential target for drug development in many diseases, including pain, inflammation, pruritus and addiction (Kieffer and Gaveriaux-Ruff, 2002; Bailey and Ribeiro-da-Silva, 2006; Chavkin, 2011; Kardon et al., 2014). Since the cloning of KOR in 1993 (Chen et al., 1993; Yasuda et al., 1993), the centrally penetrating KOR agonists have become the main focus of research for a long time. Unfortunately, although the undesirable side effects induced by mu opioid receptor (MOR) agonists are lacking, the centrally penetrating KOR agonists are frequently accompanied by certain side effects such as sedation, dysphoria and diuresis. The development of centrally penetrating KOR agonists is severely limited due to its unpleasant adverse events, and only one centrally penetrating KOR agonist nalfurafine has been approved so far for the treatment of pruritus in Japan (Inui, 2015).

To avoid these adverse side effects, other approaches have been attempted for developing the KOR agonists, and the biased KOR agonists and peripherally-restricted KOR agonists among these have gained much attention. The development of biased KOR agonists was based on the concept that G-protein coupled receptors (GPCRs) can selectively signal in different contexts (Violin et al., 2014). It is evident that opioid receptors-mediated GPCRs have the ability to interact with both G proteins and β-arrestins simultaneously. Previous studies have revealed that the side effects associated with opioid receptor activation are mediated by β-arrestin-mediated signaling pathway (Schmid et al., 2017; Hill et al., 2018b). Therefore, developing G protein-biased agonists of opioid receptors is considered as a promising strategy to bypass the CNS-mediated side effects. The biased KOR agonists were shown to be effective in treating pain and pruritus in animal models (Brust et al., 2016; Gupta et al., 2016). However, the results from two phase 3 clinical trials showed that the performance of oliceridine (TRV130), which is a biased MOR agonist, is not obviously superior to morphine, especially for reducing the respiratory depression (Singla et al., 2019; Viscusi et al., 2019). Since then, the concept of biased opioid receptor agonist has met with less enthusiasm.

The actions of peripherally-restricted KOR agonists are restricted to peripheral sites due to their low penetration into the brain, and the CNS-associated side effects associated with this can be significantly ameliorated or even completely abolished. The peripherally-restricted KOR agonists had analgesic, anti-inflammatory and antipruritic effects (Naser and Kuner, 2018). Till now, some peripherally-restricted KOR agonists, including ICI-204448, GR-94839, asimadoline, ADL-10-0116, FE200665 (CR665) and difelikefalin (CR845), have been successfully identified (Barber et al., 1994; Binder et al., 2001; Vanderah et al., 2004; Negus et al., 2012). However, the development of other compounds, except for asimadoline and CR845, has been discontinued. Originally, asimadoline was studied in treating pain and found to be ineffective. Subsequently, asimadoline has been shown to be an effective treatment for pruritus associated with atopic dermatitis (Bishop et al., 2015; Vakharia and Silverberg, 2018), but no further development of it has been reported after that. CR845, a peptide-based peripherally-restricted KOR agonist, exhibited excellent analgesic and antipruritic effects in clinical trials with limited side effects (Menzaghi et al., 2015; Fishbane et al., 2020; Steele, 2020). At present, CR845 has been approved for the treatment of moderate-to-severe pruritus associated with chronic kidney disease (CKD-aP) in adults undergoing hemodialysis. However, CR845 is only available in the United States and has some frequent adverse events, such as diarrhea, dizziness, vomiting and nasopharyngitis (Fishbane et al., 2020).

With the aim to develop more effective and safer peripherally-restricted KOR agonist, HSK21542 [7-(D-phenylalanyl-D-phenylalanyl-D-leucyl-D-lysyl)-2-acetyl-2,7-diazaspiro (3.5)nonane], was synthesized. Several studies were conducted to comprehensively address the pharmacological profiles of HSK21542. The biological activity and selectivity of HSK21542 were examined using *in vitro* assays, including [^3^H]diprenorphine binding assay, cAMP accumulation assay, and a SafetyScreen panel. Its ability to penetrate into CNS tissues was detected with a brain/plasma distribution study. Four different animal models of pain and compound 48/80-induced scratching mouse model of pruritus were used to evaluate the *in vivo* pharmacological activities of HSK21542, and the CNS side effects associated with it were also detected. Further, pharmacological profiles of HSK21542 and CR845 were compared.

## Materials and Methods

### Animals

ICR mice weighing 18–22 g and Sprague Dawley (SD) rats weighing 160–180 g were purchased from Beijing Vital River Laboratory Animal Technology Co., Ltd. (China). C57BL/6J mice weighing 18–22 g were obtained from Chengdu DOSSY Laboratory Animal Technology Co., Ltd. (China). All animals were aged between 8 and 10 weeks at the start of the experiments. Animals were maintained on a standard 12 h light/12 h dark cycle in a temperature-and humidity-controlled facility with free access to food and water. The investigators were blinded to the treatment conditions. All animal care and experimental procedures were performed in accordance with the guidelines of National Institutes of Health for the handling and use of laboratory animals and approved by the Guideline of the Institutional Animal Care and Use Committee of Haisco Pharmaceutical Group Co., Ltd. [HSK-(HEISCO-I-17)-2-1-2001-01].

### Chemicals and Reagents

**FIGURE 1:**
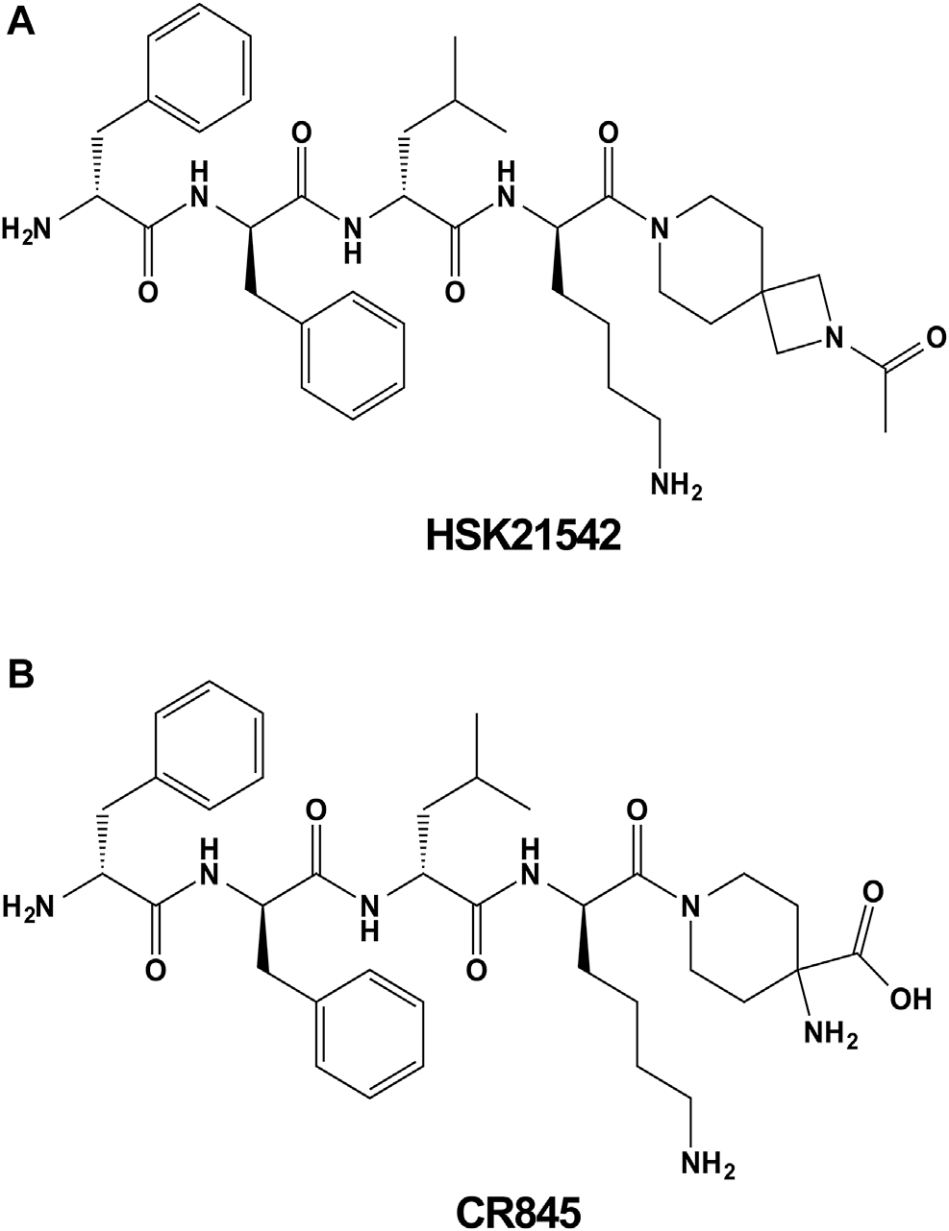
Chemical structures of HSK21542 **(A)** and CR845 **(B)**.

HSK21542 and CR845 were synthesized in Sichuan Haisco Pharmaceutical Co., Ltd. (China). The synthesis and physicochemical characteristics of HSK21542 have been described in a patent (WO2019015644). The chemical structures of HSK21542 and CR845 are shown in Figure 1. The sources of chemicals were as follows: [^3^H]diprenorphine (Perkin Elmer, United States), U69593 (Sigma-Aldrich, United States), morphine sulfate (National Institutes for Food and Drug Control, China), nor-binaltorphimine (Abcam, United Kingdom), nalfurafine hydrochloride (MedChem Express, United States) and compound 48/80 (Sigma-Aldrich, United States). For *in vivo* experiments, all the test compounds were solubilized in normal saline, and intravenously administered with a volume of 10 μL/g, except for morphine and nor-binaltorphimine (subcutaneously) when the animals were not under anesthesia and were awake. All other reagents used were of analytical grade unless otherwise stated.

### [^3^H]Diprenorphine Binding Assay

HEK-293 cells (ATCC) were maintained in Eagle’s Minimum Essential Medium with 10% FBS, and incubated at 37°C in humidified air containing 5% CO_2_. HEK-293 cells that stably express human κ opioid receptor were established in our laboratory and used in this assay. The cell membranes were prepared in 50 mM Tris-HCl buffer (pH 7.4). An equivalent of 30 μg of membranes was incubated with compounds and 0.6 nM [ ^3^ H]diprenorphine (an opioid antagonist) at 25°C for 60 min (inhibitory effect) or the multiple time points (binding kinetics). Nonspecific binding was estimated in the presence of 10 μM naloxone. The bound and free fractions were separated by vacuum filtration through a GF/B filter that was pretreated with 0.3% polyetherimide. The filters were washed with ice-cold buffer and then were counted to specifically determine the bound radioligand (Olianas et al., 2006). The percentage inhibition of [^3^H]diprenorphine binding was calculated as follows: inhibition rate (%) = (CPM_total_−CPM_compound_)/(CPM_total_−CPM_non-specific_) × 100, where CPM_total_ = total [^3^H]diprenorphine bound (membrane +0.6 nM [^3^H]diprenorphine) and CPM_non-specific_ = non-specific [^3^H]diprenorphine bound (membrane +0.6 nM [^3^H]diprenorphine + 10 μM naloxone). For the unlabeled compounds, the association/dissociation constants were calculated by fitting the data using equations as described by Motulsky and Mahan (Motulsky and Mahan, 1984).

### cAMP Accumulation Assay

PathHunter^®^ U2OS OPRK1 β-Arrestin cell line (DiscoverX) was maintained in McCoy’s 5A with 10% FBS, 250 μg/ml Hygromycin B and 500 μg/ml G418. When the cells reached to 80–90% confluence, they were collected and resuspended in HBSS (1X) containing 50 mM HEPES, 5 mM IBMX and 1% BSA stabilizer (lance^®^ UltracAMP Kit, PerkinElmer) by adjusting the cell density to 3 × 10^5^ cells/mL. The cells were divided into 384-well white plate (Corning^®^3572) at a volume of 5 μL, and were treated with compounds and 2 μM forskolin (an inducer of intracellular cAMP formation) for 30 min at room temperature. Subsequently, 5 μL of Eu-cAMP Tracer Working Solution and 5 μL of Ulight-anti-cAMP Working Solution per well were added to reach a final volume of 20 μL. The plate was incubated for 1 h at room temperature in the dark. The cAMP levels were then determined by using a microplate reader with TR-FRET assay (Wang et al., 2014). The results was expressed as (1–Signal_[compound]_/Signal_[control]_) × 100.

### *In vitro* SafetyScreen Panel

*In vitro* off-target pharmacological activities of HSK21542 were evaluated on 86 targets using a SafetyScreen panel (Item PP223, target selectivity panel) and the corresponding methods could be found at https://www.eurofinsdiscoveryservices.com/.

### *In vivo* Brain/Plasma Distribution of HSK21542 in Rats

SD rats (half male and half female) were intravenously given a single dose of 0.3 mg/kg HSK21542. The samples were collected at 0.083, 0.5, 1.5 and 4 h after dosing. The rats were anesthetized with isoflurane and then sacrificed by taking blood from the abdominal aorta. The whole brains were rapidly removed from the crania. The plasma (∼100 μL) was separated from the blood by centrifugating at 2000 ×g for 10 min at 4°C. The brains were rinsed with ice-cold normal saline, blotted dry, weighted and placed into a plastic tube. For 1.0 g of brain sample, 4 ml of acetonitrile-ultrapure water solution (1:4, v/v) was added to the tube. The brain samples were then homogenized for 120 s at 50 Hz and ultrasound was performed for 5 min. The plasma and the brain samples were analyzed using a LC-MS/MS assay as detailed in supplementary materials.

### LC-MS/MS Assay

The plasma or brain homogenate was ice thawed. After 30 μL of plasma or brain homogenate was transferred into a centrifuge tube, 50 μL of internal standard (D4-HSK21542, 50 ng/ml) and 120 μL of acetonitrile were added. The mixture was vortexed for 10 min and centrifuged at 2000 ×g for 10 min at 4°C. The collected supernatant (150 μL) was placed in a 96-well plate and dried under nitrogen. The residue was reconstituted with 150 μL of ultrapure water and vortexed for 10 min. The resulting solution was then analyzed to determine the concentrations of HSK21542 on a LC-MS/MS system, which consisted of a DGU-20A5R degasser, a LC-30AD pump, a SIL-30AC autosampler, a CTO-20A column oven (Shimadzu, Japan) and an AB Sciex Triple Quad 5500 mass spectrometer (Sciex, Canada). The LC system was coupled to mass spectrometer by using an electro-spray ionization (ESI) source (Yang et al., 2011; Dong et al., 2018). Chromatographic separation was performed on a reverse phase column (Venusil ASB C18, 4.6 mm × 50 mm) under a ternary gradient elution. The temperatures of autosampler and column were maintained at 4 and 40°C, respectively. The mobile phase A consisted of 0.3% formic acid in 2 mM acetic acid solution and the mobile phase B consisted of 0.2% formic acid in acetonitrile. The flow rate was held constant (0.7 ml/min) and the injection volume was set to 20 μL. Quantification was conducted in positive ion mode. The MRM transition of m/z 704.4→295.2 was used to quantify HSK21542.

### Writhing Test

ICR mice (half male and half female) were used in this assay. Fifteen or 30 min (only for morphine) after test compounds were given, each mouse was intraperitoneally injected with 0.6% acetic acid at a volume of 0.4 ml. Subsequently, each animal was individually maintained in a Plexiglas chamber and the pain-induced writhing behaviors were observed for 15 min (Abdollahi et al., 2003; Bourgeois et al., 2014). A writhe was defined as a wave of contraction of the abdominal musculature followed by extension of the hind limbs (Wang et al., 2018). The percentage inhibition of writhes was calculated by the following formula: % antinociception = (Nv−Nt)/Nv × 100, where Nv is the number of writhes in vehicle group and Nt is the number of writhes in treatment groups.

### Hindpaw Incision Model

The surgery was conducted as reported previously (Brennan et al., 1996; Whiteside et al., 2004; Biddlestone et al., 2007). Male SD rats were anesthetized with isoflurane inhalation (3–5%) and the plantar surface of the left hindpaw was sterilized using iodophor solution. A 1-cm longitudinal incision was made on the plantar surface with no. Eleven scalpel blade, starting at 0.5 cm from the heel and extending toward the toes. The deep fascia was cut to expose the flexor digitorum brevis muscle. The muscle was elevated with curved forceps and incised longitudinally with the tip of a scalpel blade without disturbing the origin and insertion. Following hemostasis with gentle pressure, the skin was closed with silk thread using two mattress sutures. After surgery, antibiotic ointment was applied on the incision site and the animals were returned to their home cages with clean bedding to prevent further damage to the injured hindpaw. The responses to mechanical stimulation of the hindpaws were recorded at 2 h post-surgery and the mechanical pain thresholds were defined as pre-dose values. After dividing into different groups according to the pre-dose values, the animals were administered vehicle (normal saline) or test compounds and the mechanical pain thresholds were measured at 15 min and 24 h post-dose.

### Chronic Constriction Injury Model

The CCI surgery was performed as described previously with slight modifications (Bennett and Xie, 1988). Male SD rats were used in the CCI model. After isoflurane inhalation anesthesia, the femoral skin of the left hindlimb was incised and the sciatic nerve was exposed by blunt dissection of the biceps femoris muscle with a pair of forceps (Chen et al., 2018). A 2 mm-long polyethylene cuff was successively implanted around the nerve and the incision was then closed with skin stapler (Bailey and Ribeiro-da-Silva, 2006; Balasubramanyan et al., 2006). Animals were returned to their cages after recovering from anesthesia. Seventeen days after the surgery, the responses to mechanical stimulation of the hindpaws were measured before the compounds were administered. The rats were then divided into different groups according to the pre-administration values. After the animals were given vehicle or test compounds, the mechanical pain thresholds were taken at multiple time points.

### Mechanical Allodynia Testing

The rats were placed individually in Plexiglas chambers on a metallic mesh floor and allowed to acclimatize for 30–60 min (Tsuda et al., 2011). Mechanical allodynia was determined by probing the plantar surface of the hindpaw from below the mesh floor with a series of calibrated von Frey filaments (Stoelting) in log increments of force (Lai et al., 2006). The interval between two neighboring stimulations was more than 5 s in order to eliminate the effects of the previous stimulation, and the bending angle of von Frey filaments was controlled at 15–30°. Followed by, Dixon’s up-down procedure was done to present the series of hairs and calculate the 50% paw withdrawal threshold (PWT) (Chaplan et al., 1994; Weir et al., 2017). The area under the curve (AUC, 50% PWT *vs.* time) was calculated using a trapezoid rule.

### Compound 48/80-Induced Scratching Test

After acclimatization for 30–60 min in Plexiglas chambers, the male ICR mice were given vehicle or test compounds. Compound 48/80 (50 μg, 0.1 ml) was subcutaneously injected into the back of the neck at 15 min after drug administration (Salaga et al., 2015). The mice were immediately placed back into the chambers and the scratching behaviors were recorded for 30 min (Kobayashi et al., 2003; Inan et al., 2009; Hachisuka et al., 2010). One bout of scratching was defined as the mouse lifting its hindpaw towards the injection site to scratch until it either licked or bite the hindpaw or placed it back down on the floor (Nojima and Carstens, 2003). The percentage inhibition of scratching was calculated by the following formula: % antipruritis = (Bv−Bt)/Bv × 100, where Bv is the bouts of scratching in vehicle group and Bt is the bouts of scratching in treatment groups.

### Hot-Plate Test

The hot-plate test was performed according to previous reports (DeWire et al., 2013; Hill et al., 2018a; Wang et al., 2018). Female C57BL/6J mice were individually placed on a hot plate at 56°C and the latency to licking or jumping was then recorded. A cut-off time of 30 s was imposed in order to prevent tissue damage. Before drug administration, the latency of each mouse was measured and defined as the pre-drug value. The animals with a pre-drug value of greater than 20 s were excluded. Fifteen or 30 min (only for morphine) after drug administration, the post-drug values were taken, and the percentage of maximum possible effect (% MPE) was determined as follows: [(post-drug value−pre-drug value)/(30−pre-drug value)] × 100.

### Locomotor Activity Test

The locomotor activity test was applied to analyze sedation in rats (half male and half female). Experiments were performed after animals were acclimatized to a rectangular experimental cage (35 × 35 × 35 cm^3^) for 2 days. 15 or 30 min (only for morphine) after either vehicle or test compounds were administered, each rat was returned to the cage, and then allowed to explore the field for 1 h. The data were collected using an ANY-maze video tracking system and the total distance traveled was analyzed (Gou et al., 2021).

### Measurement of Respiration

A whole body plethysmography (DSI, US) was used to measure respiration in freely moving ICR mice (half male and half female) as described previously (Manglik et al., 2016; Hill et al., 2018a; Hill et al., 2018b) with some modifications. The respiratory frequency was recorded and then averaged for over 5-min period. The baseline values were recorded for 10 min before dosing. The mice were then removed from the chambers and given drugs, and respiration was then measured for 45 min.

### Data and Statistical Analysis

All study endpoints were expressed as means ± SD and no data have been excluded. Statistical comparisons were made using GraphPad Prism 8.3.0 software (San Diego, CA, United States). No statistical methods were used to predetermine the sample sizes, but the choice of sample sizes was based on our previous studies and the sample sizes are similar to those that are typically used in the field. For *in vitro* experiments, the IC_50_ or EC_50_ value was determined by non-linear, least squares regression analysis. The binding kinetic curves were fitted by a competitive binding model. In this model, the K1 (the association rate of [^3^ H]diprenorphine) and K2 (the dissociation rate of [^3^ H]diprenorphine) were constrained to 1.44 × 10^8^ M^−1^ min^−1^ and 0.0257 min^−1^, respectively. The association kinetic curves of [^3^H]diprenorphine are shown in Supplementary Figure S1 . For most of the *in vivo* experiments, a parametric analysis (one-way analysis of variance) was performed if the Bartlett’s test for variance homogeneity showed no significance at 1% level, and the treated groups were compared to the vehicle group using Dunnett’s test when F achieves the necessary level of statistical significance (the null hypothesis: there was no difference among the treated groups and the vehicle group). Otherwise, a non-parametric analysis (Kruskal-Wallis test) was performed, and the treated groups were compared to the vehicle group using Dunn’s test when necessary. Planned comparison was done between the two groups using student’s t-test (with same variance) or Mann-Whitney test. The original data from chronic constriction injury model and the measurement of respiration in mice were analyzed by two-way analysis of variance (ANOVA) using the treatment conditions and time as factors. Then, *post-hoc* Dunnett’s test was performed at different time points if there was an interaction effect. The criterion for statistical significance was set at *p* < 0.05.

## Results

### HSK21542 is a Peripherally-Restricted Kappa Opioid Receptor Agonist

**FIGURE 2:**
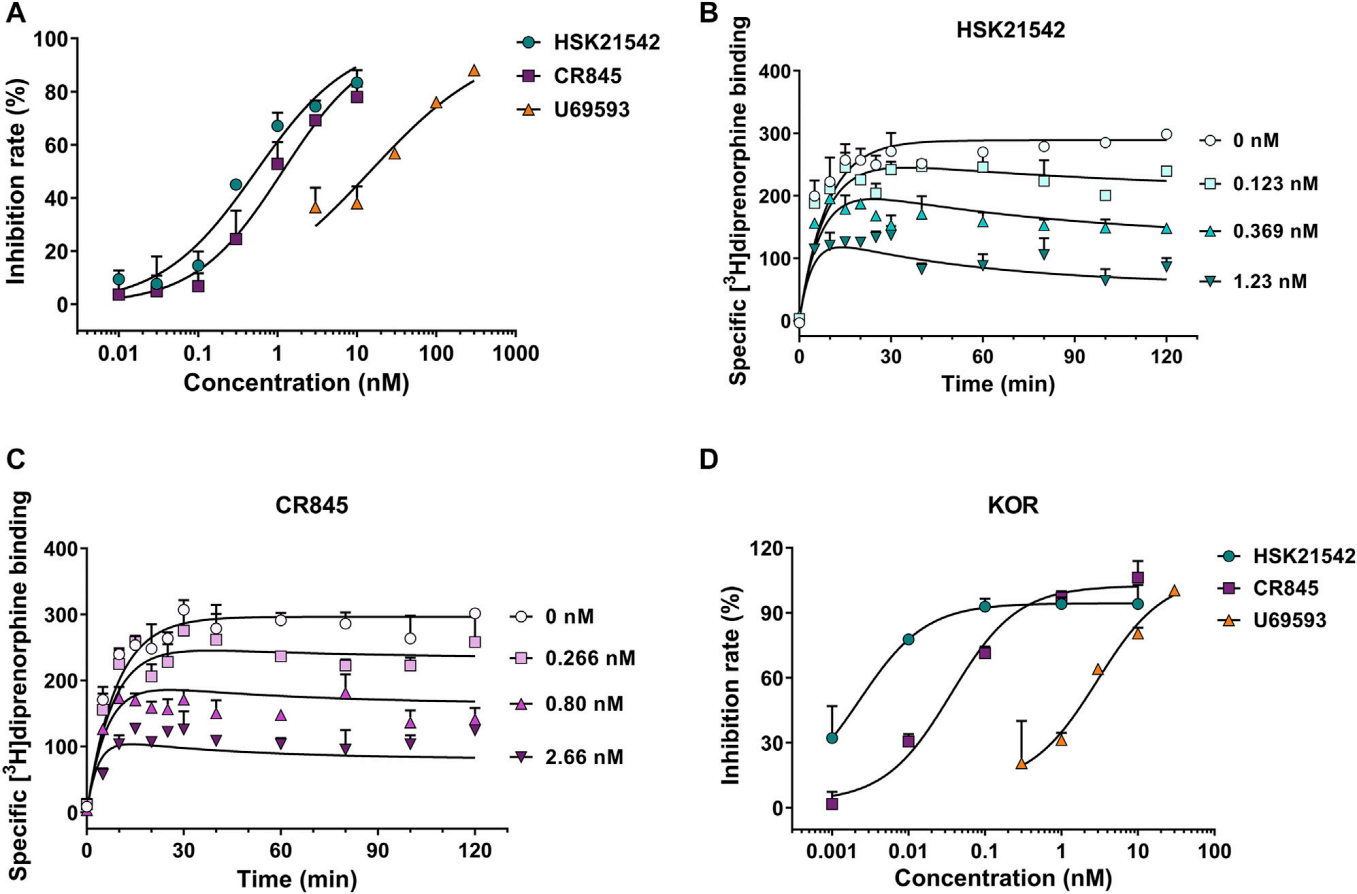
Kappa opioid receptor binding affinity and the effects on forskolin-induced cAMP accumulation of HSK21542 and CR845. **(A)** Concentration-effect curves of the inhibitory rates induced by HSK21542, CR845 or U69593 (the positive control) on [^3^H]diprenorphine binding to KOR. **(B)** The binding kinetic curves of HSK21542 binding to KOR. **(C)** The binding kinetic curves of CR845 binding to KOR. **(D)** Concentration-effect curves of the inhibitory rates induced by HSK21542, CR845 or U69593 (the positive control) on forskolin-induced cAMP accumulation. Data are presented as means ± SD of triple determinations.

To unravel the pharmacological profiles of HSK21542 at KOR, [^3^H]diprenorphine binding assay was performed to investigate the inhibitory effects of HSK21542 on [^3^H]diprenorphine competition binding and determine the binding kinetics of unlabeled HSK21542. U69593, a positive control, obviously prevented [^3^H]diprenorphine binding to KOR with an IC_50_ value of 14.72 nM (95% CI: 9.08–22.38 nM). As anticipated, HSK21542 significantly inhibited [^3^H]diprenorphine binding to KOR with an IC_50_ value of 0.54 nM (95% CI: 0.38–0.75 nM), while CR845 had an IC_50_ value of 1.16 nM (95% CI: 0.85–1.57 nM, Figure 2A). The results of the binding kinetics study revealed that HSK21542 and CR845 bound to KOR with *K*_d_ values of 0.068 nM (95% CI: 0.028–0.092 nM) and 0.23 nM (95% CI: 0.17–0.26 nM), respectively (Figures 2B,C). Meanwhile, HSK21542 had a *t*_1/2_ value of 90.6 min (95% CI: 53.6–292.7 min), which was found to be longer than that of CR845 (42.0 min, 95% CI: 28.6–79.4 min). On the other hand, HSK21542 significantly inhibited forskolin-induced cAMP accumulation in HEK-293 cells that stably expressed human κ opioid receptor with an EC_50_ value of 2.41 pM (95% CI: 1.43–4.67 pM), which was 12.4-fold and 747-fold lower than those of CR845 and U69593, respectively (Figure 2D).

To investigate the specificity, the *in vitro* profile of HSK21542 was observed against a broad panel of receptors, ion channels, transporters and enzymes, including MOR and DOR. At a concentration of 10 μM, HSK21542 was shown to bind to cannabinoid CB_1_ receptor with an inhibitory rate of 47%, and no obvious activity was observed at the remaining 85 targets (Supplementary Table S1).

Furthermore, it was extremely hard for HSK21542 to penetrate into the brain tissues with a brain/plasma concentration ratio of 0.001 (Supplementary Figure S2).

### HSK21542 Causes Potent Antinociceptive Effects

**FIGURE 3:**
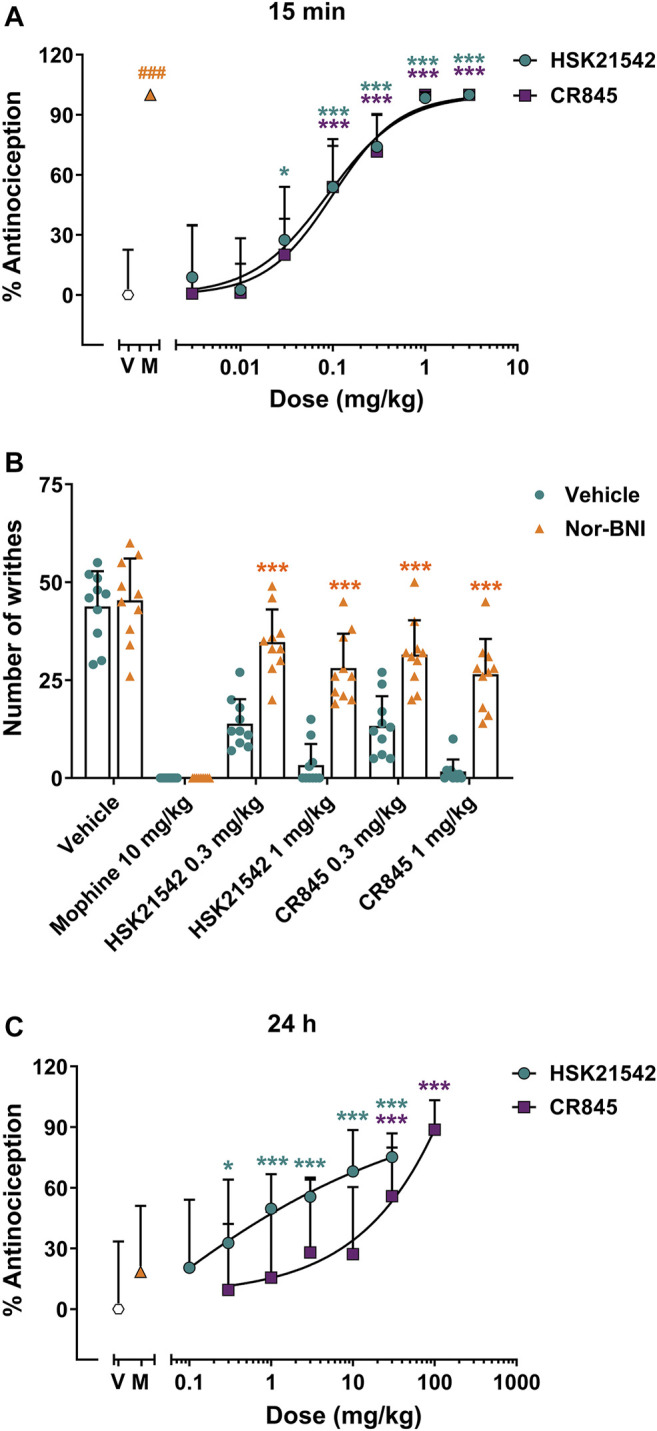
Antinociceptive effects of HSK21542 and CR845 in acetic acid-induced writhing response in mice. **(A)** The writhing tests were conducted at 15 min post-dosing. **(B)** Nor-binaltorphimine (Nor-BNI, 32 mg/kg, s.c.), a KOR antagonist, was given at 24 h before drug administration, and the antinociceptive effects of HSK21542 and CR845 were examined at 15 min post-dosing. **(C)** The writhing tests were performed at 24 h post-dosing. Data are presented as means ± SD (*n* = 10/group). **(A, C)** *p < 0.05, ***p < 0.001 vs. vehicle, one-way ANOVA followed by Dunnett’s test; **(B)** ***p < 0.001 *vs.* vehicle, Student’s t-test; ^###^ *p* < 0.001 vs. vehicle, Mann-Whitney test. V, Vehicle; M, Morphine (10 mg/kg, a positive control).

The antinociceptive effects of HSK21542 were evaluated using a writhing test in mice, which is an animal model of inflammatory pain. Both male and female mice were used to investigate any sex differences in this assay. Morphine (10 mg/kg), a positive control, completed suppressed acetic acid-induced pain behaviors at 30 min post-dosing (*p* < 0.001, Mann-Whitney test). However, the efficacy of 10 mg/kg morphine had vanished at 24 h post-dosing (t_(18)_ = 1.25, *p* = 0.30), which was corresponding to the pharmacological profile of morphine. Fifteen minutes after drug administration, HSK21542 inhibited acetic acid-induced writhing response in a dose-dependent manner (Figure 3A, F_(7, 72)_ = 41.18, *p* < 0.001), and there was no obvious sex difference (Supplementary Figure S3A, F_(1, 32)_ = 3.40, *p* = 0.075). HSK21542 at a dose of 0.03 mg/kg induced an inhibitory rate of 27.46% on writhing behaviors, and there was a statistically significant difference in writhing responses between 0.03 mg/kg HSK21542-treated group and vehicle group (*p* = 0.023). Therefore, the dose of 0.03 mg/kg was defined as the minimum effective dose (MED), which was 3.33-fold lower than that of CR845 (0.1 mg/kg). Moreover, the ED_50_ values of HSK21542 and CR845 were both 0.09 mg/kg (95% CI: 0.06–0.12 mg/kg), and the inhibitory activity of HSK21542 on writhing response was comparable to that produced by CR845 at the same doses. Finally, nor-binaltorphimine (32 mg/kg, s.c.), a kappa opioid receptor antagonist which was given at 24 h before drug administation, reversed the antinociceptive effects produced by 0.3 or 1 mg/kg HSK21542 (Figure 3B).

To explore the duration of action of a single dose of HSK21542, the antinociceptive effects of HSK21542 were detected at 24 h post-drug. Surprisingly, 0.3 mg/kg of HSK21542 still significantly inhibited the writhing responses with an inhibitory rate of 32.75% (Figure 3C, *p* = 0.02). At the doses of 1, 3, 10 and 30 mg/kg, HSK21542 induced inhibitory rates of 49.67, 55.60, 68.12 and 75.16%, respectively. However, as for CR845, a dose of 30 mg/kg was needed to maintain the antinociceptive effects at 24 h post-drug (*p* < 0.001). The ED_50_ values of HSK21542 and CR845 were 1.48 mg/kg (95% CI: 0.62–2.45 mg/kg) and 24.62 mg/kg (95% CI: 13.90–42.55 mg/kg), respectively.

### HSK21542 Produces Significant Antiallodynic Effects

The hindpaw incision model and chronic constriction injury (CCI) model in rats, which were widely used for evaluating the analgesic property, were employed to determine the antiallodynic effects of HSK21542. Morphine, a potent analgesic, was given as a positive control. Obviously, morphine (10 mg/kg) completely inhibited hindpaw incision- or CCI-induced mechanical allodynia at 15 min after dosing (*p* < 0.001, Mann-Whitney test), while morphine had no any effect on pain behaviors at 24 h post-dosing (*p* = 0.30, Mann-Whitney test).

**FIGURE 4:**
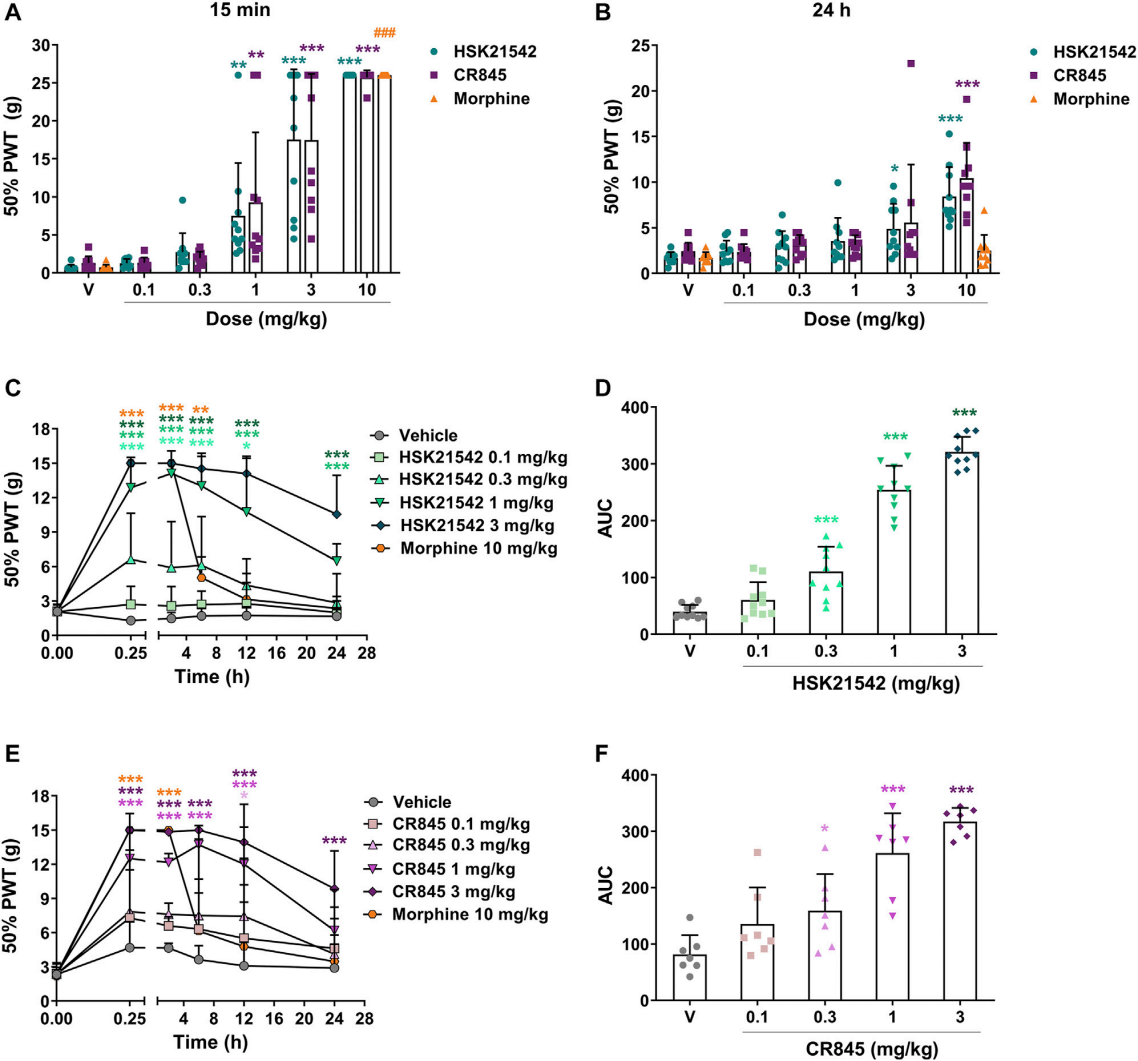
Antiallodynic effects of HSK21542 and CR845 in hindpaw incision- or CCI-induced mechanical pain. In hindpaw incision model, the mechanical allodynia testing (von Frey) was performed at 15 min **(A)** and 24 h **(B)** after drug administration. In CCI model, mechanical allodynia testing was performed at 0.25, 2, 6, 12 and 24 h post-drug **(C, E)**, and the area under the curve (AUC) was calculated using a trapezoidal method **(D, F)**. Morphine was presented as a positive control. Data are presented as means ± SD (*n* = 7–10/group). (A and B) *p < 0.05, **p < 0.01, ***p < 0.001 vs. vehicle, Kruskal-Wallis test followed by Dunn’s post-hoc test; **(C, E)** *p < 0.05, **p < 0.01, ***p < 0.001 vs. vehicle, two-way ANOVA followed by Dunnett’s test; **(D, F)** *p < 0.05, ***p < 0.001 vs. vehicle, one-way ANOVA followed by Dunnett’s test; ^###^ *p* < 0.05 vs. vehicle, Mann-Whitney test. V, Vehicle.

In the hindpaw incision model, systemic HSK21542 (0.1–10 mg/kg) exerted a dose-dependent inhibitory effect on incision-induced mechanical allodynia (*p* < 0.001, Kruskal-Wallis test). At a dose of 1 mg/kg, HSK21542 induced a 10.5-fold increase of 50% PWT (7.51 g vs. 0.72 g in the vehicle group, *p* = 0.001) at 15 min post-dosing. The MED value of HSK21542 was 1 mg/kg, which was comparable to that achieved by CR845 at 15 min after dosing, and 10 mg/kg HSK21542 induced the maximum antiallodynic activity (Figure 4A). It is noteworthy that the effects of HSK21542 were still statistically significant at 24 h after drug administration at the doses of 3 mg/kg (*p* = 0.01) and 10 mg/kg (*p* < 0.001), which were similar to those of CR845 (Figure 4B).

In the chronic constriction injury model, intravenous injection of HSK21542 (0.1–3 mg/kg) suppressed CCI-induced mechanical allodynia in rats in a dose-dependent manner (F_(5, 316)_ = 245.0, *p* < 0.001). At a dose of 0.3 mg/kg, HSK21542 induced a 5.15-fold increase of 50% PWT (6.62 g vs. 1.29 g in the vehicle group) at 15 min post-dosing. In 1 or 3 mg/kg HSK21542-treated group, the 50% PWT value reached a peak at 2 h post-dosing, and then gradually faded (Figure 4C). Furthermore, when given at a dose of 1 or 3 mg/kg, the mechanical pain threshold in HSK21542-treated groups was significantly higher than that in the vehicle-treated group at 24 h post-dosing. The results showed that the treatment with 0.3 mg/kg HSK21542 induced a statistically significant increase on the AUC value (50% PWT vs. time, Figure 4D, *p* < 0.001). Therefore, the dose of 0.3 mg/kg was defined as the MED value of HSK21542. On the other hand, there was no obvious difference in mechanical pain thresholds between 0.1 mg/kg HSK21542-treated group and vehicle-treated group at any time points post-dosing. Moreover, although the antiallodynic effects of CR845 could still persist until 24 h post-administration at a dose of 3 mg/kg (*p* < 0.001), the effects of CR845 had completely vanished at a dose of 1 mg/kg (Figure 4E, *p* = 0.13).

### HSK21542 Attenuates Compound 48/80-Induced Itch

**FIGURE 5:**
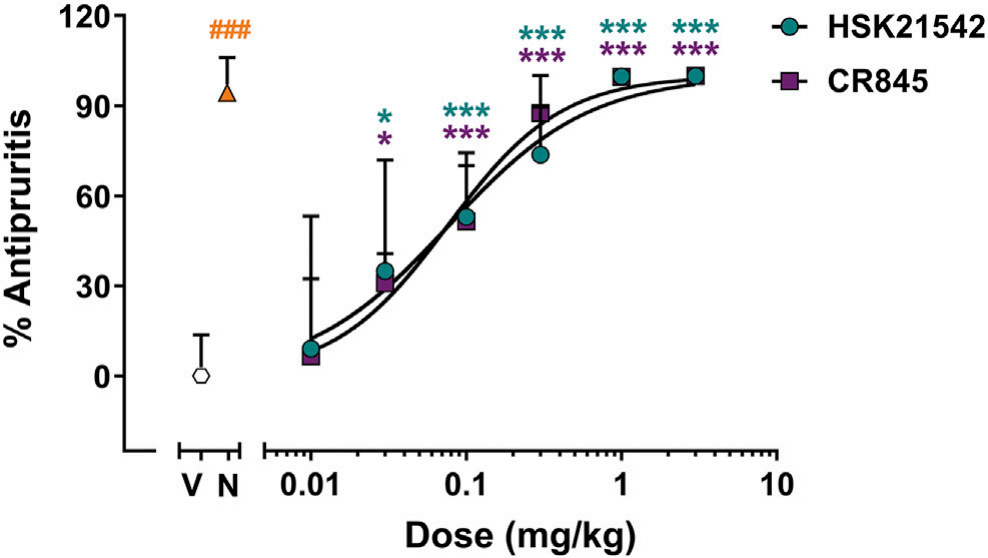
Antipruritic effects of HSK21542 and CR845 in compound 48/80-induced itch model. Data are presented as means ± SD (*n* = 10/group). *p < 0.05, ***p < 0.001 vs. vehicle, one-way ANOVA followed by Dunnett’s test; ^###^ *p* < 0.001 vs. vehicle, Mann-Whitney test. V, Vehicle; N, Nalfurafine (0.02 mg/kg, a positive control).

KOR agonist has been validated as an effective therapy for pathological itch (Kardon et al., 2014; Cowan et al., 2015; Inui, 2015). The antipruritic effects of HSK21542 were then determined with compound 48/80-induced scratching test in mice. In the compound 48/80-induced scratching test, when given at a dose of 0.02 mg/kg, nalfurafine (a positive control) effectively suppressed the scratching responses with an inhibitory rate of 94.30% (*p* < 0.001, Mann-Whitney test). HSK21542 (0.01–3 mg/kg) inhibited the scratching responses to a similar extent as did CR845 in a dose-dependent manner at 15 min post-drug (Figure 5, F_(6, 63)_ = 27.82, *p* < 0.001). At a dose of 0.03 mg/kg, HSK21542 induced an inhibitory rate of 34.89%, and the number of scratching bouts was statistically less than that in the vehicle-treated group (*p* = 0.015). Therefore, the MED value of HSK21542 was designated as 0.03 mg/kg. At a dose of 1 mg/kg, the antipruritic activity of HSK21542 reached a peak with an inhibitory rate of 99.78%. In the 0.1 and 0.3 mg/kg HSK21542-treated groups, the inhibitory rates of 53.02 and 73.75% were observed, respectively. Furthermore, the analysis of dose-response curve showed that HSK21542 had an ED_50_ value of 0.09 mg/kg (95% CI: 0.04–0.16 mg/kg), and this was comparable to that of CR845 (0.10 mg/kg, 95% CI: 0.04–0.23 mg/kg, *p* = 0.91, Extra sum-of-squares F test).

### HSK21542 Showed Fewer CNS Side Effects

**FIGURE 6:**
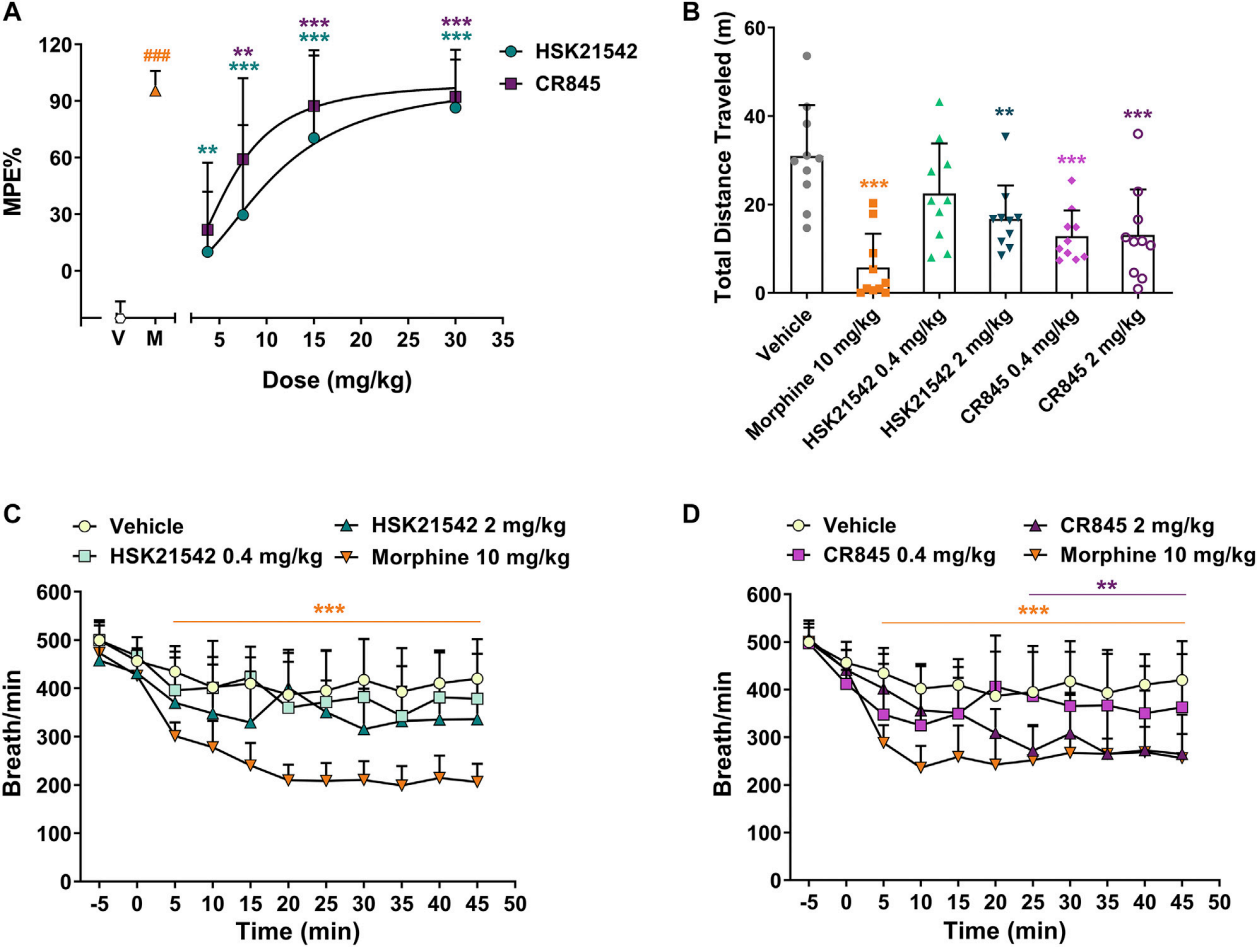
Central antinociceptive activities, and effects on locomotor activity and respiratory rate of HSK21542 and CR845 in mice. **(A)** The central antinociceptive activities were evaluated using a hot-plate test in mice at 15 min post-drug. **(B)** The sedative effects at 15 min post-dosing were detected using a locomotor activity test. **(C, D)** The effects on respiratory rate in mice were measured with whole body plethysmography. Data are presented as means ± SD (*n* = 10/group). **(A, B)** **p < 0.01, ***p < 0.001 vs. vehicle, one-way ANOVA followed by Dunnett’s test; **(C, D)** **p < 0.01, ***p < 0.001 vs. vehicle, two-way ANOVA followed by Dunnett’s test; ^###^ *p* < 0.001 vs. vehicle, Mann-Whitney test. V, Vehicle; M, Moprhine (10 mg/kg, a positive control).

As a supraspinal model for acute pain, the hot-plate test is considered as one of the experimental methods for differentiating the central and peripheral antinociceptive effects (Le Bars et al., 2001). To validate whether the *in vivo* pharmacological effects of HSK21542 are mediated by a peripheral mechanism, a hot-plate test was employed in mice to evaluate the central antinociceptive effects of HSK21542 at 15 min post-drug. As one of central analgesics, 10 mg/kg morphine (a positive control) showed an almost complete efficacy. HSK21542 at 3.75 mg/kg did not evoke significant antinociceptive effects (*p* = 0.12), although 7.5 mg/kg HSK21542 induced a percent maximum possible effect (% MPE) of 29.60%, which was statistically higher than that in the vehicle-treated group (*p* = 0.007). However, CR845 displayed significant antinociceptive effects at a dose of 3.75 mg/kg (Figure 6A, *p* = 0.008). In addition, the effects of HSK21542 at 7.5 mg/kg were comparable to that of CR845 at 3.75 mg/kg (29.60% *vs.* 21.67%, t_(18)_ = 0.42, *p* = 0.68). The ED_50_ values of HSK21542 and CR845 were 10.49 mg/kg (95% CI: 7.58–15.37 mg/kg) and 6.76 mg/kg (95% CI: 4.70–8.69 mg/kg), respectively (*p* = 0.026, Extra sum-of-squares F test).

To directly explore the CNS effects of HSK21542, its effects on sedation and respiration rate were measured. The sedative effects of HSK21542 and CR845 at 15 min post-dosing were evaluated using a locomotor activity test. The results showed that 10 mg/kg morphine reduced remarkedly the distance traveled by mice (*p* < 0.001) and there was no sex difference (Supplementary Figure S4, F_(1, 24)_ = 4.19, *p* = 0.052). Although HSK21542 at 2 mg/kg induced an obvious sedative effect (*p* = 0.005), the lower dose of HSK21542 (0.4 mg/kg) did not significantly affect the locomotor activity of mice (Figure 6B, *p* = 0.16). On the other hand, 0.4 mg/kg CR845 induced an obvious decrease in the total distance traveled by mice (*p* < 0.001) with comparable efficacy to the higher dose of HSK21542 or CR845 (*p* > 0.05, Student’s t-test). As shown in Figure 6C, 10 mg/kg morphine significantly reduced the respiratory rate (*p* < 0.001). There were no obvious effects on respiration when HSK21542 was given at a dose of as high as 2 mg/kg (*p* > 0.05). In contrast, CR845 caused significant decrease in the respiratory rate at a dose of 2 mg/kg at 25 min post-administration and the effects reached a peak at 45 min post-drug (Figure 6D, *p* < 0.01).

## Discussion

The current work provided significant findings, validating that HSK21542 is a peripherally-restricted KOR agonist and has an outstanding translational potential. These studies revealed that the combination of radioligand [^3^H]diprenorphine and KOR was significantly inhibited by HSK21542, which could bind to KOR with high affinity, and HSK21542 inhibited cAMP accumulation induced by KOR activation. On the other hand, HSK21542 had no obvious biological effects on the remaining 86 targets. Meanwhile, the brain/plasma concentration ratio of HSK21542 remained extremely low, suggesting its peripheral selectivity. Furthermore, HSK21542 produced powerful analgesic and antipruritic effects in animal models. HSK21542 attenuated acetic acid-induced writhing response and the therapeutic efficacy was maintained for over 24 h by a single intravenous dose of 0.1–3 mg/kg. Meanwhile, the analgesic activity of HSK21542 could be reversed by the KOR antagonist nor-BNI, indicating its on-target activity. HSK21542 also suppressed hindpaw incision- or CCI-induced mechanical allodynia and the effects were still able to be detected at 24 h post-drug within a certain range of doses. Moreover, HSK21542 inhibited compound 48/80-induced scratching response. Finally, HSK21542 lacked obvious antinociceptive effects in hot-plate test, and had weaker effects on the locomotor activity and respiratory rate in mice.

CR845 is a peripherally-restricted KOR agonist that has been originally developed by Ferring Pharmaceuticals SA, and has been approved for treating pruritus. To validate if HSK21542 would be a better alternative for patients who are suffering from pain or pruritus, its pharmacological profiles were compared to those of CR845. In [^3^H]diprenorphine binding assay, the IC_50_ and *K*_d_ values of HSK21542 were much smaller than those of CR845, and HSK21542 had a longer *t*_1/2_ value for disassociating from KOR. These results indicated that HSK21542 acts as a ligand of KOR with higher affinity. Moreover, HSK21542 reversed forskolin-induced cAMP accumulation in HEK-293 cells that stably express human κ opioid receptor with subnanomolar potency, and was superior to CR845.

In 0.6% acetic acid-induced writhing test, HSK21542 induced potent antinociceptive effects at 15 min after systemic administration. The profile of HSK21542 was similar to that of CR845. Although the ED_50_ value of HSK21542 was completely identical to that of CR845 at 15 min post-dose, the ED_50_ value of HSK21542 was 16.6-fold lower than that of CR845 at 24 h post-drug. In addition, the MED of HSK21542 was 3.33–100 times lower than that of CR845. Therefore, HSK21542 is considered as a more promising candidate than CR845 for treating pain. To verify this conclusion, the antiallodynic effects of HSK21542 and CR845 were also evaluated in a hindpaw incision model and a CCI model. As predicted, HSK21542 achieved outstanding antiallodynic effects, which were comparable or even superior to CR845. The more potent analgesic effects of HSK21542 are attributed to its excellent *in vitro* biological activities. It is undeniable that HSK21542 might be a more potent and longer-acting analgesic than CR845. To further address the *in vivo* pharmacological profiles of HSK21542 and CR845, their antipruritic activities were measured in an animal model of compound 48/80-induced itch, wherein HSK21542 presented remarkable antipruritic effects, similar to that of CR845.

To validate HSK21542 as a safer candidate drug for treating pain and pruritus, the central antinociceptive effects of HSK21542 and CR845 were assessed with a hot-plate test, and the effects on locomotor activity and respiration in mice were observed. In the hot-plate test, the MED value (7.5 mg/kg) of HSK21542 was higher than the dose (1 mg/kg) needed to produce maximum antinociceptive effects in writhing test. The ED_50_ value of HSK21542 was 10.49 mg/kg, suggesting that HSK21542 has a therapeutic index of 116.6 (Supplementary Table S2). In contrast, CR845 has a smaller therapeutic index (75.1). Furthermore, HSK21542 at a dose of 2 mg/kg did not affect the locomotor activity and respiratory rate in mice, which was obviously higher than the doses needed to produce analgesic and antipruritic effects. However, CR845 suppressed the respiratory rate in mice at the same dose. Morphine, a typical representative of MOR agonist, highly inhibited the respiratory rate in mice at a dose of 10 mg/kg, at which the antinociceptive effects of morphine was comparable to that achieved by HSK21542 or CR85 (Supplementary Figure S5). Therefore, HSK21542 does not produce obvious CNS effects, which are typical profiles of centrally penetrating KOR agonists and MOR agonist. In view of all these results, HSK21542 might have a larger translational potential than CR845.

However, it is noteworthy that there is a huge challenge in preclinical-to-clinical translation for analgesic and antipruritic candidates. One of the main causes is the discordance in endpoints between animal and human studies. In animals, the pain- or itch-stimulated behaviors are recorded to label analgesic or antipruritic candidates, making it unavoidable that the false-positive effects might exist, resulting from non-selective drug effects such as sedation and paralysis (Lazenka et al., 2018). Therefore, it is important to look forward to the results of clinical trials that will validate if HSK21542 could be a safe and effective analgesic and antipruritic drug. At present, HSK21542 is under Phase II clinical development for treating pain or pruritis (CTR20201702, CTR20201210, CTR20200371; http://www.chinadrugtrials.org.cn/clinicaltrials.searchlist.dhtml).

In conclusion, the *in vitro* findings revealed that HSK21542 is a selective KOR agonist with a higher potency than CR845. The brain/plasma distribution study showed that HSK21542 has an extremely poor ability to penetrate into the CNS system. The *in vivo* pharmacological activities supported the translational potential of HSK21542 as a safe and effective analgesic and antipruritic candidate. Generally, HSK21542 has the ability to avoid adverse CNS effects that are associated with centrally penetrating KOR agonists and MOR agonist, and might provide an effective alternative for treating patients with pain or pruritus.

## Data Availability

The original contributions presented in the study are included in the article/Supplementary Material, further inquiries can be directed to the corresponding authors.

## Abbreviations

BSA, bovine serum albumin; CCI, chronic constriction injury; CI, onfidence interval; CNS, central nervous system; DOR, delta opioid receptor; EC_50_, half maximal effective concentration; ED_50_, median effective dose; FBS, fetal bovine serum; GPCR, G-protein coupled receptor; HBSS, hank’s balanced salt solution; HEPES, hydroxyethyl piperazineethanesulfonic acid; IBMX, 3-Isobutyl-1-methylxanthine; IC_50_, 50% inhibitory concentration; KOR, kappa opioid receptor; MED, minimum effective dose; MOR, mu opioid receptor; MPE, maximum possible effect; PWT, paw withdrawal threshold; SD, standard deviation; TR-FRET, time-resolved fluorescence resonance energy transfer.
