# Supplementary material for: Antinociceptive and Antipruritic Effects of HSK21542, a Peripherally-Restricted Kappa Opioid Receptor Agonist, in Animal Models of Pain and Itch
Source: Front Pharmacol. 2021 Nov 16;12:773204. doi: 10.3389/fphar.2021.773204 (PMC8635029; doi:10.3389/fphar.2021.773204)
Supplement: Supplementary file 5 [file Table1.DOC]

**Table S1**. *In vitro* off-target pharmacological profile of HSK21542

| **Assay Name** | **Species** | **Conc. (μM)** | **Inhibition (%)** |
| --- | --- | --- | --- |
| Adenosine A_1_ | human | 10 | 0 |
| Adenosine A_2A_ | human | 10 | 3 |
| Adrenergic α_1A_ | rat | 10 | 12 |
| Adrenergic α_1B_ | rat | 10 | 3 |
| Adrenergic α_1D_ | human | 10 | -1 |
| Adrenergic α_2A_ | human | 10 | 7 |
| Adrenergic α_2B_ | human | 10 | -6 |
| Adrenergic β_1_ | human | 10 | 11 |
| Adrenergic β_2_ | human | 10 | 3 |
| Androgen (Testosterone) | human | 10 | 3 |
| Angiotensin AT_1_ | human | 10 | -7 |
| ATPase, Na+/K+, Heart, Pig | pig | 10 | 0 |
| Bradykinin B_2_ | human | 10 | -3 |
| Calcium Channel L-Type, Benzothiazepine | rat | 10 | 12 |
| Calcium Channel L-Type, Dihydropyridine | rat | 10 | -11 |
| Calcium Channel L-Type, Phenylalkylamine | rat | 10 | 13 |
| Calcium Channel N-Type | rat | 10 | -3 |
| Cannabinoid CB_1_ | human | 10 | 47 |
| Cannabinoid CB_2_ | human | 10 | -30 |
| Chemokine CCR1 | human | 10 | -4 |
| Chemokine CXCR2 (IL-8R_B_) | human | 10 | 4 |
| Cholecystokinin CCK_1_ (CCK_A_) | human | 10 | 5 |
| Cholecystokinin CCK_2_ (CCK_B_) | human | 10 | -12 |
| Cholinesterase, Acetyl, ACES | human | 10 | 6 |
| Cyclooxygenase COX-1 | human | 10 | 9 |
| Cyclooxygenase COX-2 | human | 10 | 12 |
| Dopamine D_1_ | human | 10 | 3 |
| Dopamine D_2L_ | human | 10 | -6 |

**Table S1**. *In vitro* off-target pharmacological profile of HSK21542 (continuing)

| **Assay Name** | **Species** | **Conc. (μM)** | **Inhibition (%)** |
| --- | --- | --- | --- |
| Dopamine D_2S_ | human | 10 | -15 |
| Endothelin ET_A_ | human | 10 | 4 |
| Estrogen ERα | human | 10 | 0 |
| GABA_A_, Chloride Channel, TBOB | rat | 10 | -22 |
| GABA_A_, Flunitrazepam, Central | rat | 10 | -10 |
| GABA_A_, Ro-15-1788, Hippocampus | rat | 10 | 5 |
| GABA_B1A_ | human | 10 | -8 |
| Glucocorticoid | human | 10 | 8 |
| Glutamate, AMPA | rat | 10 | -2 |
| Glutamate, Kainate | rat | 10 | 3 |
| Glutamate, Metabotropic, mGlu_5_ | human | 10 | 19 |
| Glutamate, NMDA, Agonism | rat | 10 | 2 |
| Glutamate, NMDA, Glycine | rat | 10 | -1 |
| Glutamate, NMDA, Phencyclidine | rat | 10 | 10 |
| Glutamate, NMDA, Polyamine | rat | 10 | 2 |
| Glycine, Strychnine-Sensitive | rat | 10 | -6 |
| Histamine H_1_ | human | 10 | 4 |
| Histamine H_2_ | human | 10 | 8 |
| Leukotriene, Cysteinyl CysLT_1_ | human | 10 | 8 |
| Melanocortin MC_1_ | human | 10 | -3 |
| Melanocortin MC_4_ | human | 10 | 27 |
| Monoamine Oxidase MAO-A | human | 10 | 0 |
| Monoamine Oxidase MAO-B | human | 10 | 11 |
| Muscarinic M_1_ | human | 10 | 9 |
| Muscarinic M_2_ | human | 10 | 1 |
| Muscarinic M_3_ | human | 10 | -3 |
| Muscarinic M_4_ | human | 10 | 3 |
| Neuropeptide YY_1_ | human | 10 | -8 |

**Table S1**. *In vitro* off-target pharmacological profile of HSK21542 (continuing)

| **Assay Name** | **Species** | **Conc. (μM)** | **Inhibition (%)** |
| --- | --- | --- | --- |
| Nicotinic Acetylcholine | human | 10 | -9 |
| Nicotinic Acetylcholine α1, Bungarotoxin | human | 10 | -5 |
| Opiate δ1 (OP1, DOP) | human | 10 | 5 |
| Opiate μ (OP3, MOP) | human | 10 | 0 |
| Peptidase, Angiotensin Converting Enzyme | rabbit | 10 | 5 |
| Peptidase, CTSG (Cathepsin G) | human | 10 | -1 |
| Phosphodiesterase PDE3 | human | 10 | -4 |
| Phosphodiesterase PDE4 | human | 10 | 12 |
| Platelet Activating Factor (PAF) | human | 10 | 10 |
| Potassium Channel [K_ATP_] | human | 10 | 1 |
| Potassium Channel hERG | human | 10 | 1 |
| PPARγ | human | 10 | 3 |
| Progesterone PR-B | human | 10 | -1 |
| Protein Serine/Threonine Kinase, PKC, Non-Selective | rat | 10 | 5 |
| Protein Tyrosine Kinase, Insulin Receptor | human | 10 | 17 |
| Protein Tyrosine Kinase, LCK | human | 10 | -8 |
| Serotonin (5-Hydroxytryptamine) 5-HT_1A_ | human | 10 | 1 |
| Serotonin (5-Hydroxytryptamine) 5-HT_1B_ | human | 10 | 1 |
| Serotonin (5-Hydroxytryptamine) 5-HT_2A_ | human | 10 | 0 |
| Serotonin (5-Hydroxytryptamine) 5-HT_2B_ | human | 10 | 0 |
| Serotonin (5-Hydroxytryptamine) 5-HT_2C_ | human | 10 | 0 |
| Serotonin (5-Hydroxytryptamine) 5-HT_3_ | human | 10 | 14 |
| Sodium Channel, Site 2 | rat | 10 | -19 |
| Tachykinin NK_1_ | human | 10 | 21 |
| Transporter, Adenosine | guinea pig | 10 | -5 |
| Transporter, Dopamine (DAT) | human | 10 | 2 |
| Transporter, GABA | rat | 10 | 0 |

**Table S1**. *In vitro* off-target pharmacological profile of HSK21542 (continuing)

| **Assay Name** | **Species** | **Conc. (μM)** | **Inhibition (%)** |
| --- | --- | --- | --- |
| Transporter, Norepinephrine (NET) | human | 10 | 19 |
| Transporter, Serotonin (5-Hydroxytryptamine) (SERT) | human | 10 | 7 |
| Vasopressin V1A | human | 10 | -24 |

**Table S2.** Therapeutic indexes of HSK21542 and CR845.

|  | **Compound** | |
| --- | --- | --- |
|  | **HSK21542** | **CR845** |
| Writhing test, ED_50_ (mg/kg) | 0.09 | 0.09 |
| Hot-plate test, ED_50_ (mg/kg) | 10.49 | 6.76 |
| Therapeutic index | 116.6 | 75.1 |
